# Supplementary material for: Aggregation of rhodopsin mutants in mouse models of autosomal dominant retinitis pigmentosa
Source: Nat Commun. 2024 Feb 16;15:1451. doi: 10.1038/s41467-024-45748-4 (PMC10873427; doi:10.1038/s41467-024-45748-4)
Supplement: Supplementary file 1 — Supplementary Information [file 41467_2024_45748_MOESM1_ESM.pdf]

Supplementary Information

Aggregation of rhodopsin mutants in mouse models of autosomal dominant retinitis pigmentosa

Sreelakshmi Vasudevan, Subhadip Senapati, Maryanne Pendergast, and Paul S.–H. Park

Department of Ophthalmology and Visual Sciences, Case Western Reserve University, Cleveland, OH 44106, USA

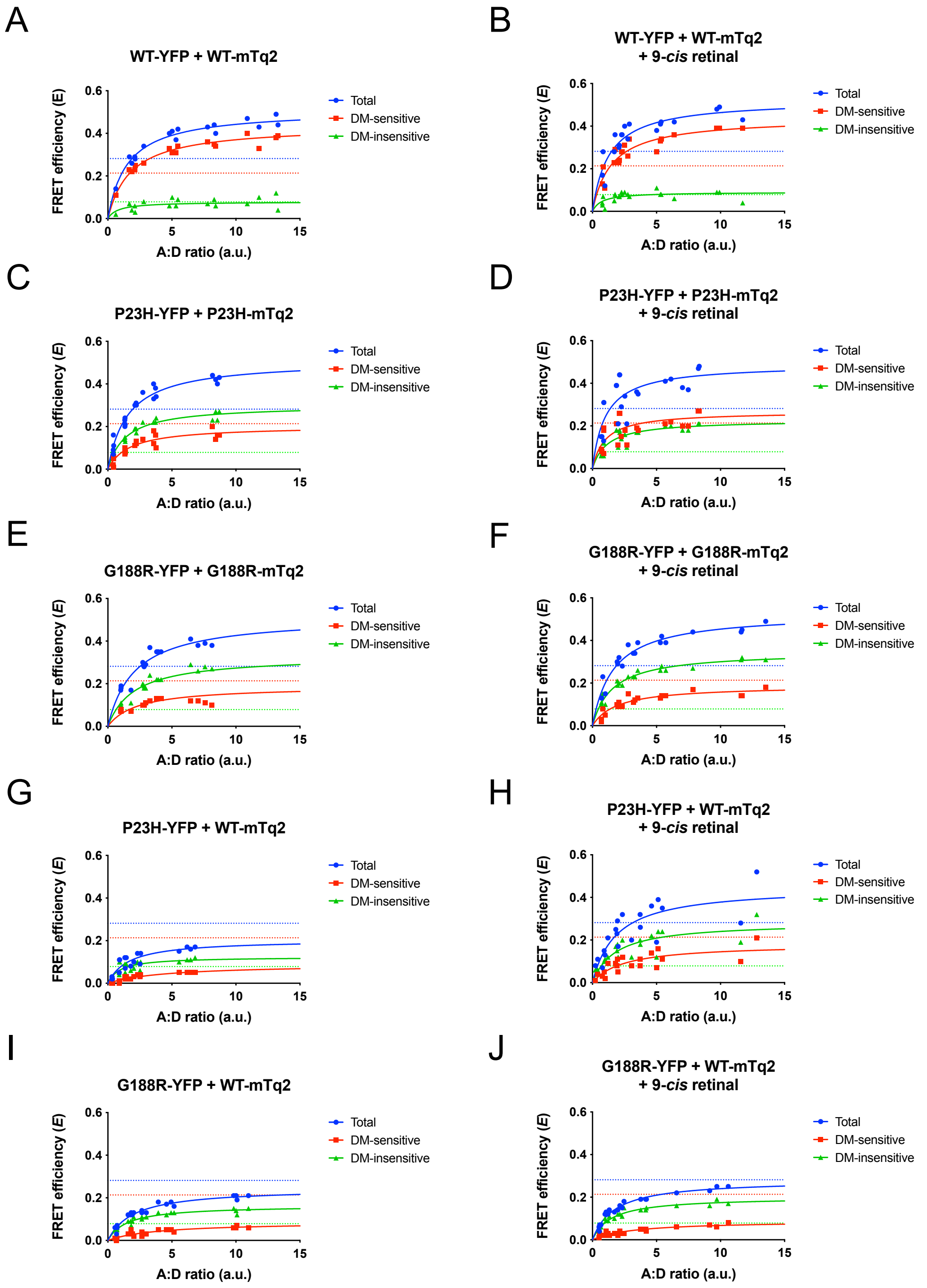

Supplementary Figure 1. FRET curves. FRET curves were generated from cells expressing the indicated YFP-tagged and mTq2-tagged WT, P23H, or G188R murine rhodopsin. Cells were either untreated (A, C, E, G, I) or treated with 15 μM 9-*cis* retinal (B, D, F, H, J). Total (blue), DM-sensitive (red), and DM-insensitive (green) FRET curves are shown. Each curve contains data from 4 separate experiments, which were simultaneously fit with a rectangular hyperbolic function. Fitted lines are shown and values obtained from fits are reported in Fig. 2 and Supplementary Table 1. The non-specific FRET E_{max}, defined previously [1], is indicated by the dashed lines.

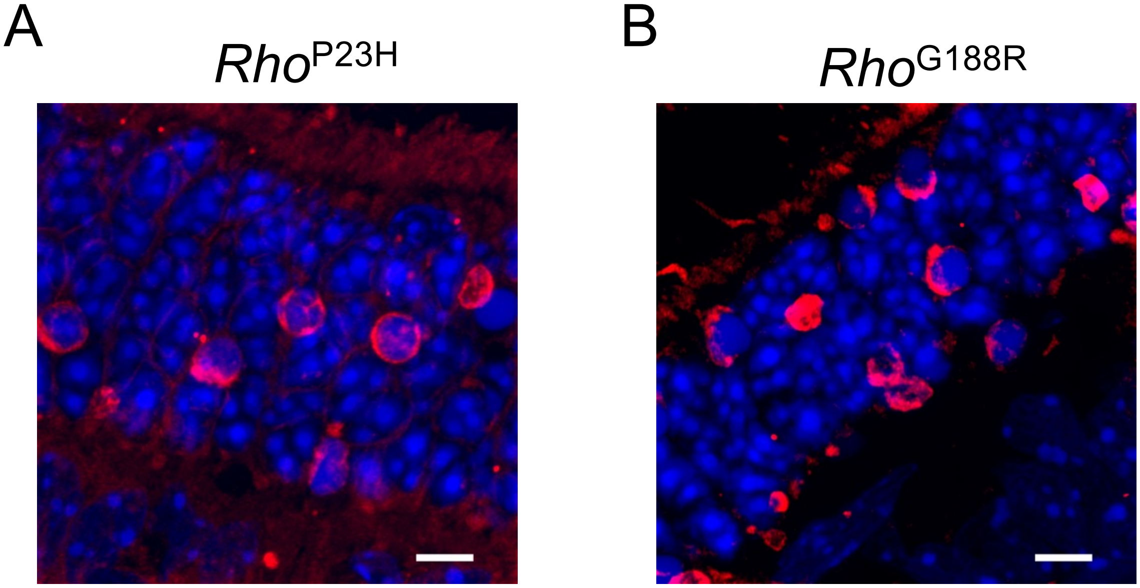

Supplementary Figure 2. High magnification maximum intensity projection images of PROTEOSTAT staining in the outer nuclear layer of homozygous mutant mice. Retina cryosections from 2-week-old *Rho*^{P23H} (A) and *Rho*^{G188R} (B) mice were stained with PROTEOSTAT (red), and nuclei were stained with NucBlue (blue). Scale bar, 5 µm. Images are representative of at least 3 different experiments.

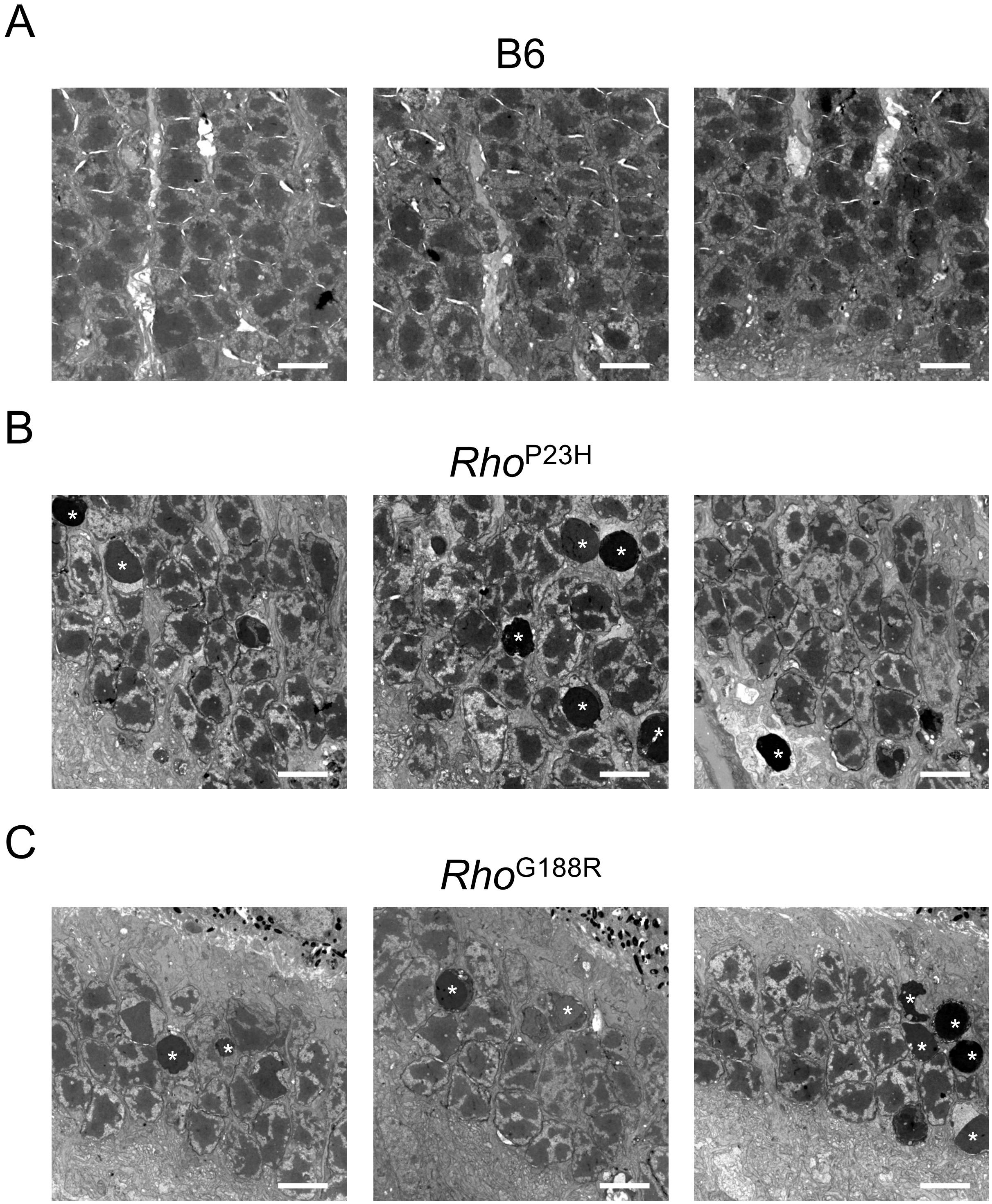

Supplementary Figure 3. Electron microscopy of outer nuclear layer. EM images of thin sections of retina in the outer nuclear layer of 2-week-old B6 (A), *Rho*^{P23H} (B), and *Rho*^{G188R} (C) mice are shown. Nuclei with condensed chromatin, indicative of a dying cell, are highlighted by asterisk (*). Scale bar, 5 μm. Images are representative of at least 3 different experiments.

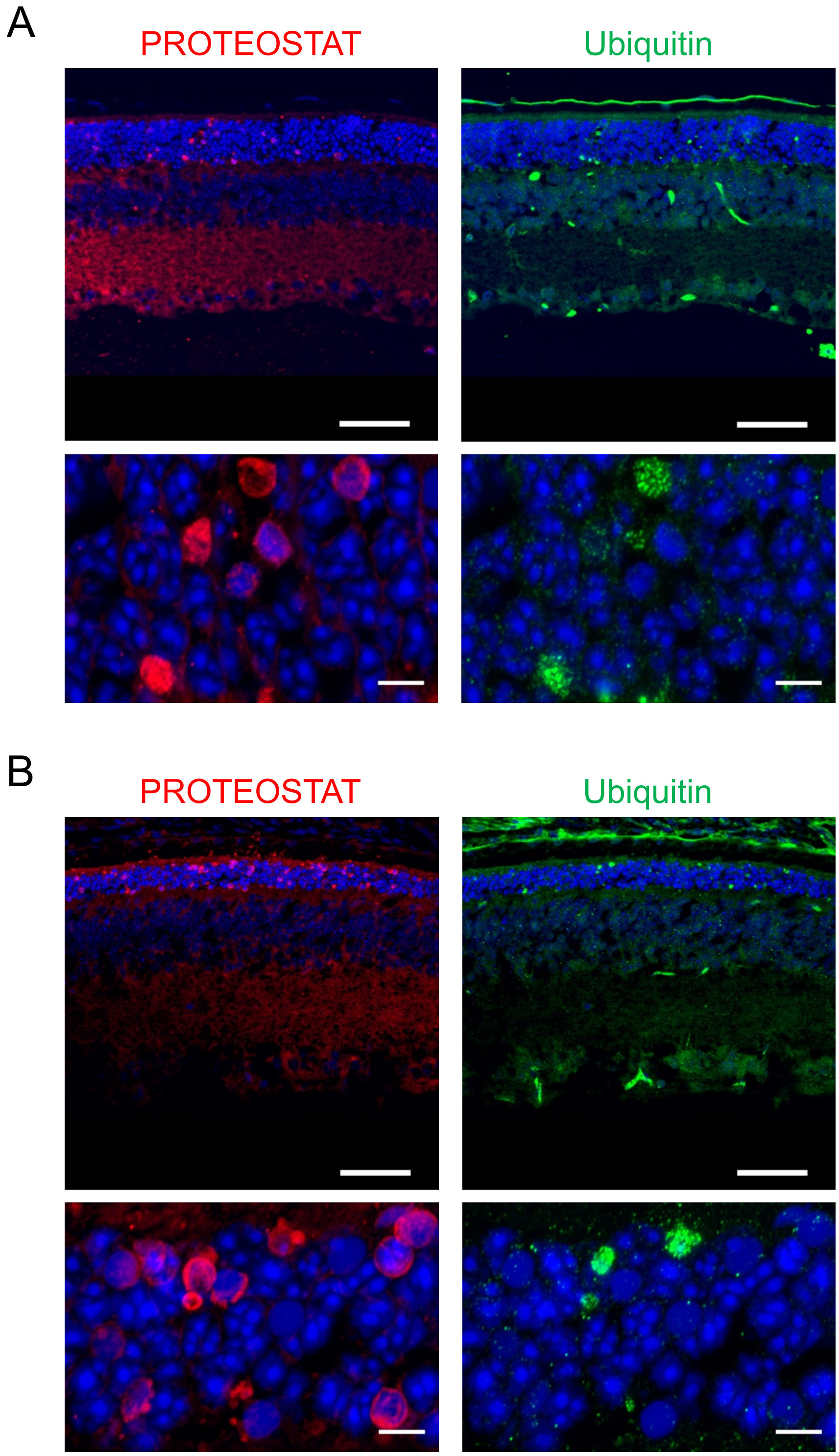

Supplementary Figure 4. PROTEOSTAT and anti-ubiquitin antibody staining. Merged images in Fig. 8B were separated to show the individual signals from PROTEOSTAT (red) and anti-ubiquitin antibody (green) staining for *Rho*^{P23H} (A) and *Rho*^{G188R} (B) mice. NucBlue staining is shown in blue. Top images, scale bar, 50 µm. Bottom images, scale bar, 5 µm. Images are representative of at least 3 different experiments.

Supplementary Table 1. FRET curve analysis

Coexpressed receptors and treatment	Parameter					
	E_{max}			EC_{50}		
	**Total**	**DM-sensitive**	**DM-insensitive**	**Total**	**DM-sensitive**	**DM-insensitive**
**WT-YFP + WT-mTq2**	0.51 ± 0.01	0.44 ± 0.01	0.08 ± 0.01	1.53 ± 0.15	1.79 ± 0.19	0.79 ± 0.73
**WT-YFP + WT-mTq2 + 9-*cis* retinal**	0.52 ± 0.03	0.44 ± 0.02	0.09 ± 0.01	1.28 ± 0.25	1.49 ± 0.26	0.71 ± 0.40
**P23H-YFP + P23H-mTq2**	0.51 ± 0.02	0.20 ± 0.02	0.30 ± 0.01	1.58 ± 0.20	1.70 ± 0.47	1.40 ± 0.17
**P23H-YFP + P23H-mTq2 + 9-*cis* retinal**	0.49 ± 0.03	0.27 ± 0.02	0.23 ± 0.01	1.02 ± 0.28	0.98 ± 0.33	1.16 ± 0.24
**G188R-YFP + G188R-mTq2**	0.51 ± 0.02	0.19 ± 0.02	0.33 ± 0.01	2.09 ± 0.24	2.31 ± 0.60	2.05 ± 0.27
**G188R-YFP + G188R-mTq2 + 9-*cis* retinal**	0.53 ± 0.02	0.19 ± 0.01	0.35 ± 0.01	1.64 ± 0.22	1.93 ± 0.46	1.58 ± 0.17
**P23H-YFP + WT-mTq2**	0.20 ± 0.02	0.09 ± 0.02	0.12 ± 0.01	1.63 ± 0.45	4.63 ± 1.58	0.94 ± 0.34
**P23H-YFP + WT-mTq2 + 9-*cis* retinal**	0.45 ± 0.06	0.18 ± 0.03	0.28 ± 0.04	1.88 ± 0.71	2.48 ± 1.14	1.78 ± 0.62
**G188R-YFP + WT-mTq2**	0.24 ± 0.01	0.09 ± 0.01	0.16 ± 0.01	2.00 ± 0.25	3.99 ± 1.22	1.50 ± 0.23
**G188R-YFP + WT-mTq2 + 9-*cis* retinal**	0.28 ± 0.01	0.09 ± 0.01	0.20 ± 0.01	1.71 ± 0.18	3.24 ± 0.91	1.48 ± 0.24

Data in Supplementary Fig. 1 was fit to a rectangular hyperbolic function as described in the Methods. Fitted values for the maximal FRET efficiency (E_{max}) and EC_{50} are shown along with the standard errors.

Supplementary Table 2. Extra sum of squares *F* test analysis of FRET data

Coexpressed receptors and treatment	*P*-value		
	**Total**	**DM-sensitive**	**DM-insensitive**
**WT-YFP + WT-mTq2**	<0.0001 (F (1,18) = 825.7)	<0.0001 (F (1,18) = 840.0)	0.9661 (F (1,18) = 0.001859)
**WT-YFP + WT-mTq2 + 9-*cis* retinal**	<0.0001 (F (1,18) = 128.4)	<0.0001 (F (1,18) = 179.5)	0.2310 (F (1,18) = 1.537)
**P23H-YFP + P23H-mTq2**	<0.0001 (F (1,18) = 235.5)	0.5885 (F (1,18) = 0.3035)	<0.0001 (F (1,18) = 1146)
**P23H-YFP + P23H-mTq2 + 9-*cis* retinal**	<0.0001 (F (1,23) = 62.95)	0.0117 (F (1,23) = 7.508)	<0.0001 (F (1,23) = 308.6)
**G188R-YFP + G188R-mTq2**	<0.0001 (F (1,18) = 320.6)	0.1850 (F (1,18) = 1.900)	<0.0001 (F (1,18) = 1085)
**G188R-YFP + G188R-mTq2 + 9-*cis* retinal**	<0.0001 (F (1,17) = 275.4)	0.1216 (F (1,17) = 2.656)	<0.0001 (F (1,17) = 1653)
**P23H-YFP + WT-mTq2**	0.0178 (F (1,18) = 6.801)	0.0057 (F (1,18) = 9.831)	0.0007 (F (1,18) = 16.91)
**P23H-YFP + WT-mTq2 + 9-*cis* retinal**	0.0017 (F (1,18) = 13.50)	0.4195 (F (1,18) = 0.6827)	<0.0001 (F (1,18) = 116.6)
**G188R-YFP + WT-mTq2**	0.0056 (F (1,18) = 9.883)	0.0004 (F (1,18) = 18.47)	<0.0001 (F (1,18) = 274.3)
**G188R-YFP + WT-mTq2 + 9-*cis* retinal**	0.9635 (F (1,18) = 0.002155)	0.0005 (F (1,18) = 18.14)	<0.0001 (F (1,18) = 274.4)

Data in Figs. 2A-2C and Supplementary Fig. 1 were analyzed by an extra sum of squares *F* test to test whether or not each E_{max} is different from the non-specific FRET E_{max} values of 0.28, 0.21, and 0.078 for total, DM-sensitive, and DM-insensitive FRET, respectively. *P*-values are reported with the *F*-statistic shown in parentheses.

Supplementary Table 3. Summary of aggregation profiles of WT, P23H, and G188R rhodopsin

Rhodopsin mutant	Expressed alone		Coexpressed with WT	
	Untreated	9-*cis* retinal	Untreated	9-*cis* retinal
**P23H**	Aggregates	Aggregates and oligomers	No complex with WT	Aggregates with WT
**G188R**	Aggregates	Aggregates	No complex with WT	No complex with WT

Supplementary Table 4. ERG analysis in Figs. 4G-4I

Mouse	Scotopic a-wave		Scotopic b-wave				Photopic b-wave	
	R_{max} (μV)	log K_A	R_{max} (μV)	log K_A	log K_B	f	R_{max} (μV)	log K_A
**B6**	78 ± 2	-1.02 ± 0.07	244 ± 9	-3.12 ± 0.17	-0.37 ± 0.32	0.65 ± 0.05	79 ± 4	0.50 ± 0.08
Rho$^{P23H/+}$	49 ± 1	-0.99 ± 0.06	234 ± 9	-2.62 ± 0.13	-0.21 ± 0.35	0.68 ± 0.06	87 ± 4	0.47 ± 0.07
Rho$^{G188R/+}$	25 ± 1	-1.08 ± 0.07	134 ± 5	-2.61 ± 0.19	-0.48 ± 0.27	0.55 ± 0.07	60 ± 2	0.54 ± 0.06

Data in Figs. 4G-4I were fit to dose-response models described in the Methods. Fitted values are shown along with the standard errors.

Supplementary Table 5. Extra sum of squares F test analysis of ERG data

Comparisons	*P*-value for comparison tests of shared parameters						
	Scotopic a-wave		Scotopic b-wave			Photopic b-wave	
	R_{max}	log K_A	R_{max}, log K_B, f	log K_B, f	log K_A, log K_B, f	R_{max}	log K_A
**B6 vs *Rho***$^{P23H/+}$	<0.0001 (F (1, 248) = 117.3)	0.7899 (F (1, 248) = 0.07117)	0.5157 (F (3, 244) = 0.7632)	0.9298 (F (2, 244) = 0.07281)	0.0265 (F (3, 244) = 3.126)	0.1184 (F (1, 192) = 2.461)	0.7990 (F (1, 192) = 0.06504)
**B6 vs *Rho***$^{G188R/+}$	<0.0001 (F (1, 257) = 116.5)	0.7629 (F (1, 257) = 0.09117)	<0.0001 (F (3, 253) = 76.32)	0.6802 (F (2, 253) = 0.3860)	0.0062 (F (3, 253) = 4.224)	0.0002 (F (1, 199) = 14.48)	0.7271 (F (1, 199) = 0.1221)
Rho$^{P23H/+}$ **vs *Rho***$^{G188R/+}$	<0.0001 (F (1, 239) = 313.1)	0.4074 (F (1, 239) = 0.6889)	<0.0001 (F (3, 235) = 81.84)	0.5016 (F (2, 235) = 0.5016)	0.3147 (F (3, 235) = 1.189)	<0.0001 (F (1, 185) = 35.33)	0.5081 (F (1, 185) = 0.4397)

Data in Figs. 4G-4I were analyzed by an extra sum of squares F test to test whether or not the indicated shared parameters were different among the mouse lines. *P*-values are reported with the F-statistic shown in parentheses.

Supplementary Table 6. *P*-values for one-way ANOVA and Dunnett's post-hoc analysis

	P-value				
	ANOVA	*Rho*$^{P23H/+}$	*Rho*$^{G188R/+}$	*Rho*^{P23H}	*Rho*^{G188R}
***18s rRNA* normalization**	<0.0001 (F (4, 79) = 160.2)	0.3125	0.1496	<0.0001	<0.0001
***Gnat1* normalization**	<0.0001 (F (4, 79) = 12.85)	0.6213	0.6714	<0.0001	<0.0001

Statistical analyses were conducted on data in Fig. 5A. Data from each mutant mouse line was compared to that from the B6 control by Dunnett's post-hoc analysis. *P*-values are reported with the *F*-statistic shown in parentheses for the ANOVA analysis.

Supplementary Table 7. ROS disc properties from AFM analysis of mice 4 weeks of age

ROS disc property	Parameter value		
	Rho$^{P23H/+}$ (n = 53)	[a]B6 (n = 77)	[a]*Rho*$^{+/-}$ (n = 52)
**Disc diameter (µm)**	1.17 ± 0.39	1.21 ± 0.23	1.09 ± 0.25
**Median nanodomain size (nm^2)**	716 ± 250	1,079 ± 283	988 ± 258
**Number of nanodomains**	128 ± 95	145 ± 79	86 ± 43
**Nanodomain density (µm^{-2})**	221 ± 68	206 ± 72	146 ± 58
**Number of rhodopsin**	10711 ± 8307	15610 ± 9219	8867 ± 4685
**Rhodopsin density (µm^{-2})**	17917 ± 4423	21654 ± 8484	14672 ± 5489

Mean values along with the standard deviation are reported. The number of images analyzed is given by *n*. Data are presented in graphical form in Figs. 6C-6H and statistical analyses of the data are reported in Supplementary Table 8.

[a] These data are included as a reference and were those that were reported previously [2].

Supplementary Table 8. *P*-values for one-way ANOVA and Tukey's post-hoc analysis of AFM data

	***P*-value**					
	**Disc diameter**	**Median nanodomain size**	**Number of nanodomains**	**Nanodomain density**	**Number of rhodopsin**	**Rhodopsin density**
**ANOVA**	0.0636 (F (2, 179) = 2.798)	<0.0001 (F (2, 179) = 29.83)	0.0001 (F (2, 179) = 9.470)	<0.0001 (F (2, 179) = 18.66)	<0.0001 (F (2, 179) = 12.74)	<0.0001 (F (2, 179) = 17.23)
***Rho*$^{\mathbf{P23H/+}}$ vs B6**	0.7415	<0.0001	0.4391	0.4289	0.0018	0.0058
***Rho*$^{\mathbf{P23H/+}}$ vs *Rho*$^{+/-}$**	0.2982	<0.0001	0.0136	<0.0001	0.4569	0.0370
**B6 vs *Rho*$^{+/-}$**	0.0512	0.1408	<0.0001	<0.0001	<0.0001	<0.0001

Statistical analyses were conducted on data in Figs. 6C-6H and Supplementary Table 7. *P*-values are shown for ANOVA and Tukey's post-hoc analysis for individual comparisons. The *F*-statistic is shown in parentheses for the ANOVA analysis.

References

1 Gragg, M. & Park, P. S. Misfolded rhodopsin mutants display variable aggregation properties. *Biochim. Biophys. Acta* **1864**, 2938-2948 (2018). https://doi.org:10.1016/j.bbadis.2018.06.004

2 Rakshit, T. & Park, P. S. Impact of reduced rhodopsin expression on the structure of rod outer segment disc membranes. *Biochemistry* **54**, 2885-2894 (2015). https://doi.org:10.1021/acs.biochem.5b00003
