# Supplementary material for: Aggregation of rhodopsin mutants in mouse models of autosomal dominant retinitis pigmentosa
Source: Nat Commun. 2024 Feb 16;15:1451. doi: 10.1038/s41467-024-45748-4 (PMC10873427; doi:10.1038/s41467-024-45748-4)
Supplement: Supplementary file 3 — Description of Additional Supplementary Files [file 41467_2024_45748_MOESM3_ESM.pdf]

Description of Additional Supplementary Files

Supplementary Movie 1. 3D reconstruction of nucleus in first image shown in Figure 8D. DAPI staining is shown in blue and PROTEOSTAT staining is shown in red.

Supplementary Movie 2. 3D reconstruction of nucleus in second image shown in Figure 8D. DAPI staining is shown in blue and PROTEOSTAT staining is shown in red.

Supplementary Movie 3. 3D reconstruction of nucleus in third image shown in Figure 8D. DAPI staining is shown in blue and PROTEOSTAT staining is shown in red.

Supplementary Movie 4. 3D reconstruction of nucleus in fourth image shown in Figure 8D. DAPI staining is shown in blue and PROTEOSTAT staining is shown in red.

Supplementary Movie 5. 3D reconstruction of nuclei in fifth and sixth images shown in Figure 8D. DAPI staining is shown in blue and PROTEOSTAT staining is shown in red.

Supplementary Movie 6. 3D reconstruction of nucleus in seventh image shown in Figure 8D. DAPI staining is shown in blue and PROTEOSTAT staining is shown in red.
