# Supplementary material for: Aggregation of rhodopsin mutants in mouse models of autosomal dominant retinitis pigmentosa
Source: Nat Commun. 2024 Feb 16;15:1451. doi: 10.1038/s41467-024-45748-4 (PMC10873427; doi:10.1038/s41467-024-45748-4)
Supplement: Supplementary file 10 — Reporting Summary [file 41467_2024_45748_MOESM10_ESM.pdf]

Corresponding author(s): Paul S.-H. Park

Last updated by author(s): Jan 24, 2024

Reporting Summary

Nature Portfolio wishes to improve the reproducibility of the work that we publish. This form provides structure for consistency and transparency in reporting. For further information on Nature Portfolio policies, see our Editorial Policies and the Editorial Policy Checklist.

Statistics

For all statistical analyses, confirm that the following items are present in the figure legend, table legend, main text, or Methods section.

n/a	Confirmed	
☐	☒	The exact sample size (*n*) for each experimental group/condition, given as a discrete number and unit of measurement
☐	☒	A statement on whether measurements were taken from distinct samples or whether the same sample was measured repeatedly
☐	☒	The statistical test(s) used AND whether they are one- or two-sided<br>*Only common tests should be described solely by name; describe more complex techniques in the Methods section.*
☒	☐	A description of all covariates tested
☒	☐	A description of any assumptions or corrections, such as tests of normality and adjustment for multiple comparisons
☐	☒	A full description of the statistical parameters including central tendency (e.g. means) or other basic estimates (e.g. regression coefficient) AND variation (e.g. standard deviation) or associated estimates of uncertainty (e.g. confidence intervals)
☐	☒	For null hypothesis testing, the test statistic (e.g. *F*, *t*, *r*) with confidence intervals, effect sizes, degrees of freedom and *P* value noted<br>*Give P values as exact values whenever suitable.*
☒	☐	For Bayesian analysis, information on the choice of priors and Markov chain Monte Carlo settings
☒	☐	For hierarchical and complex designs, identification of the appropriate level for tests and full reporting of outcomes
☐	☒	Estimates of effect sizes (e.g. Cohen's *d*, Pearson's *r*), indicating how they were calculated

Our web collection on statistics for biologists contains articles on many of the points above.

Software and code

Policy information about availability of computer code

Data collection	FluoroMax-4 spectrofluorometer, FluorEssence Version 2.5.2.0 (Horiba Jobin Yvon, Edison, NJ)<br>SP8 confocal microscope, LAS X Version 3.5.5.19976 (Leica, Buffalo Grove, IL)<br>Olympus FV1200 confocal microscope, FV10-ASW 4.2 and cellSens Dimension 4.2 (Evident/Olympus, Waltham, MA)<br>Axio Scan.Z1 Slide Scanner, Zen 3.1 (blue version) (Carl Zeiss Microscopy, White Plains, NY)<br>Celeris rodent ERG system, Espion 6.0 (Diagnosys, Lowell, MA)<br>Odyssey Fc Imaging System, LI-COR Image Studio Version 4 (LI-COR Biosciences, Lincoln, NE)<br>LightCycler 96 Real-Time PCR System, LightCycler 96 version 1.1 (Roche Diagnostics, Indianapolis, IN)<br>Multimode II atomic force microscope, Nanoscope 5.3 (Bruker, Santa Barbara, CA)<br>Tecnai G2 Spirit Bio electron microscope (FEI company, Hillsboro, OR) with Gatan Rio 16 camera, Digital Micrograph version 3.44.3448.0 (Gatan, Pleasanton, CA)
Data analysis	Prism 9 (GraphPad Software, San Diego, CA)<br>Fiji (version 2.1.0/1.53c)<br>ImageJ (version 1.53n)<br>SPIP (version 6.7, Image Metrology A/S, Hørsholm, Denmark)<br>MetaMorph Version 7.7.8.0 (Molecular Devices, San Jose, CA)<br>Huygens Professional 23.10 (Scientific Volume Imaging, Hilversum, the Netherlands)

For manuscripts utilizing custom algorithms or software that are central to the research but not yet described in published literature, software must be made available to editors and reviewers. We strongly encourage code deposition in a community repository (e.g. GitHub). See the Nature Portfolio guidelines for submitting code & software for further information.

Data

Policy information about availability of data

All manuscripts must include a data availability statement. This statement should provide the following information, where applicable:

- Accession codes, unique identifiers, or web links for publicly available datasets
- A description of any restrictions on data availability
- For clinical datasets or third party data, please ensure that the statement adheres to our policy

All data supporting the findings of this study are available within the paper and in supplementary information files. The crystal structure of rhodopsin presented in Fig. 1B is from the Protein Data Bank (PDB ID: 1U19). Raw graphical data and uncropped Western blots are included in a source data file. Source data are provided with this paper.

Research involving human participants, their data, or biological material

Policy information about studies with human participants or human data. See also policy information about sex, gender (identity/presentation), and sexual orientation and race, ethnicity and racism.

Reporting on sex and gender	No human participants or data were used
Reporting on race, ethnicity, or other socially relevant groupings	No human participants or data were used
Population characteristics	No human participants or data were used
Recruitment	No human participants or data were used
Ethics oversight	No human participants or data were used

Note that full information on the approval of the study protocol must also be provided in the manuscript.

Field-specific reporting

Please select the one below that is the best fit for your research. If you are not sure, read the appropriate sections before making your selection.

☒ Life sciences ☐ Behavioural & social sciences ☐ Ecological, evolutionary & environmental sciences

For a reference copy of the document with all sections, see nature.com/documents/nr-reporting-summary-flat.pdf

Life sciences study design

All studies must disclose on these points even when the disclosure is negative.

Sample size	No sample size calculation was performed a priori, and sample sizes used were based on previous studies. The number of samples tested was equal to or greater than sample size calculations for differences of up to 20% that have 80 % power with type 1 error of 5 % (computed using Eq. 3 in Dziak et al. (2020) Curr. Psychol. 39, 870-877).
Data exclusions	No data was excluded.
Replication	All quantitative analyses provide the number of samples or mice tested. All attempts at replication were successful. Qualitative studies were conducted on at least 3 different samples or mice to confirm reproducibility.
Randomization	Treatments were not used in the current study and therefore randomization procedures were not necessary. All mice were housed in the same room and experienced the same husbandry. All samples from mice were prepared by the same investigator.
Blinding	The husbandry of mice is performed irrespective of the mouse line and therefore all mice are treated equally. Blinding was not performed because the same investigator prepared samples, performed experiments and analyzed the data.

Reporting for specific materials, systems and methods

We require information from authors about some types of materials, experimental systems and methods used in many studies. Here, indicate whether each material, system or method listed is relevant to your study. If you are not sure if a list item applies to your research, read the appropriate section before selecting a response.

Materials & experimental systems

n/a	Involved in the study	
☐	☒	Antibodies
☐	☒	Eukaryotic cell lines
☒	☐	Palaeontology and archaeology
☐	☒	Animals and other organisms
☒	☐	Clinical data
☒	☐	Dual use research of concern
☒	☐	Plants

Methods

n/a	Involved in the study	
☒	☐	ChIP-seq
☒	☐	Flow cytometry
☒	☐	MRI-based neuroimaging

Antibodies

Antibodies used

anti-1D4, 1:500 for IHC or 1:2500 for WB (Molday and Mackenzie (1983) Biochemistry 22, 653-660)
anti-4D2, 1:1000, MilliporeSigma (Burlington, MA), Cat. No. MABN15, Lot No. 3674950
anti-ubiquitin (P4D1 clone), 1:100, Santa Cruz Biotechnology (Dallas, TX), Cat. No. sc-8017, Lot No. G1423
anti-GAPDH, 1:5000, Proteintech (Rosemont, IL), Cat. No. 10494-1-AP, Lot No. 00130019
CF 647 goat anti-mouse secondary antibody, 1:500, MilliporeSigma (Burlington, MA), Cat. No. SAB4600183, Lot No. 22C1026
IRDye 800CW Donkey anti-Mouse IgG, 1:4000, LI-COR Biosciences (Lincoln, NE), Cat. No. 926-32212, Lot No. D21109-15
IRDye 680LT Donkey anti-Rabbit IgG, 1:4000, LI-COR Biosciences (Lincoln, NE), Cat. No. 925-68023, Lot No. D20419-11

Validation

All commercial antibodies were validated by the vendor for the species and methods used. Additionally, we made sure that there were previously published studies using these antibodies for similar purposes.
anti-1D4 antibody: This is a well characterized and widely used antibody that was originally validated in Molday and Mackenzie (1983) Biochemistry 22, 653-660.
anti-4D2 antibody: Manufacturer indicates reactivity for human, rat, fish, and mouse species in immunohistochemistry, Western blot, immunocytochemistry, and ELISA applications. Our staining of retinal sections from rhodopsin knockout mice in the current study confirms the specificity of the antibody.
anti-ubiquitin antibody: Manufacturer indicates reactivity for human, rat, mouse, and Drosophila melanogaster species in immunohistochemistry, Western blot, immunoprecipitation, immunofluorescence, flow cytometry, and ELISA applications. Over 3000 publications cited using this product.
anti-GAPDH antibody: Manufacturer indicates reactivity for human, rat, mouse, pig, Arabidopsis, cor cabbage, and rice species in immunohistochemistry, Western blot, immunoprecipitation, immunofluorescence, flow cytometry, and ELISA applications. Over 5000 publications cited using this product.

Eukaryotic cell lines

Policy information about cell lines and Sex and Gender in Research

Cell line source(s)

HEK293T/17 cells (Cat. No. CRL-11268, American Type Culture Collection, Manassas, VA)

Authentication

Cell line validated by vendor using STR profiling

Mycoplasma contamination

Cells were free from Mycoplasma contamination

Commonly misidentified lines
(See ICLAC register)

No commonly misidentified cell lines were used

Animals and other research organisms

Policy information about studies involving animals; ARRIVE guidelines recommended for reporting animal research, and Sex and Gender in Research

Laboratory animals

Mouse: C57Bl/6J (stock no. 000664, The Jackson Laboratory (Bar Harbor, ME)), RhoP23H (stock no. 017628, The Jackson Laboratory (Bar Harbor, ME)), Prph2Rd2 (stock no. 001979, The Jackson Laboratory (Bar Harbor, ME)). Rho-/- mice were provided by Dr. Paul Sieving (UC Davis, Sacramento, CA) and are on a predominantly C57Bl/6J background. RhoG188R mice were generated in the current study by CRISPR/Cas9 and are on a C57Bl/6J background. Mice aged 2 weeks to 6 months of age were examined. All animal studies reported here were conducted using protocols approved by the Institutional Animal Care and Use Committee at Case Western Reserve University School of Medicine. Mice were housed in rooms maintained at 22 degrees C and 50 % humidity under cyclic 12 h dark/12h light. Mice were euthanized by carbon dioxide inhalation.

Wild animals

No wild animals were used.

Reporting on sex

No apparent sex differences were observed in preliminary assessments and therefore sex was not considered in the analyses of the data. Both male and female mice were included in experiments, however, experiments were not designed to examine sex-specific differences. Thus, data were not disaggregated for sex.

Field-collected samples	No field-collected samples were used.
Ethics oversight	Animal studies were approved by the Institutional Animal Care and Use Committee (IACUC) at Case Western Reserve University School of Medicine (Animal Welfare Assurance Number A3145-01) under protocol 2014-0021.

Note that full information on the approval of the study protocol must also be provided in the manuscript.
